# Sustained High Levels of Both Total and High Molecular Weight Adiponectin in Plasma during the Convalescent Phase of Haemorrhagic Fever with Renal Syndrome Are Associated with Disease Severity

**DOI:** 10.1155/2017/6468097

**Published:** 2017-03-23

**Authors:** Kang Tang, Chunmei Zhang, Yusi Zhang, Yun Zhang, Ran Zhuang, Boquan Jin, Ying Ma

**Affiliations:** Department of Immunology, The Fourth Military Medical University, Xi'an 710032, China

## Abstract

Haemorrhagic fever with renal syndrome (HFRS) is characterised by an uncontrolled immune response that causes vascular leakage. Adiponectin (APN) is an adipocytokine involved in prorevascularisation and immunomodulation. To investigate the possible effects of APN in the pathogenesis of HFRS, total and high molecular weight (HMW) APN levels in the plasma of patients with HFRS were quantified using enzyme-linked immunosorbent assay (ELISA). Compared with those in healthy controls, the plasma total and HMW APN levels in patients were elevated to different degrees from the fever onset and remained high at the convalescent phase. Consistent with these results, western blot analysis additionally showed that low molecular weight (LMW), middle molecular weight (MMW), and HMW APN levels were all elevated and contributed to the elevation of the total APN level. Importantly, sustained high levels of total and HMW APN at the convalescent phase were significantly higher in patients with critical disease than those in patients with mild or moderate disease. Moreover, total and HMW APN levels negatively correlated with white blood cell count and positively correlated with platelet count and serum albumin level. These results may provide insights into understanding the roles of total and HMW APN in the pathogenesis of HFRS.

## 1. Introduction

Hantaan virus (HTNV) infection can cause severe haemorrhagic fever with renal syndrome (HFRS) in humans. HFRS is an infectious disease of global concern characterised by increased vascular endothelial permeability, thrombocytopenia, and renal damage [1]. China is one of the most severe endemic areas of HFRS in the world, with the highest incidence of approximately 100,000 cases each year [2]. HTNV infection of endothelial cells (ECs) and HTNV-induced immunological activations of the complement system and cytokine storm, such as increased proinflammatory cytokines interleukin (IL)-1, IL-6, and tumor necrosis factor-*α* (TNF-*α*), could induce the reorganization of the endothelial cytoskeleton and junctions and mediate an increase in EC permeability [1, 3]. Enhanced EC permeability leads to the dysfunction of the EC barrier, manifested as petechia, oedema, and hypotension, which may underlie the pathogenesis of HFRS [4]. Increased thrombopoiesis and platelet activation may induce intravascular coagulation and cause thrombocytopenia, the accumulation of inflammatory cells, and the release of proinflammatory cytokines in kidney tissue contributing to renal damage [4, 5].

Adiponectin (APN) is a 244-amino acid glycoprotein predominantly secreted by adipocytes and other cells such as lymphocytes, bone-forming cells, and epithelial cells. APN circulates in human plasma at high concentrations of 0.5–30 *μ*g/mL, which is approximately 0.01% of total plasma protein [6, 7]. Plasma APN exists in different biologically active isoforms, including the ~68 kDa trimer (low molecular weight, LMW), the ~150 kDa hexamer (middle molecular weight, MMW), and the >250 kDa 12-mer or higher (high molecular weight, HMW) APN. Some of the LMW APNs can bind to albumin (ALB) to form a complex (ALB-LMW) [6, 8, 9]. Importantly, the various forms of APN do not interconvert in the serum [6]. Four types of receptors for APN have been identified, including ADIPOR1, ADIPOR2, T-cadherin, and calreticulin/CD91. By binding these receptors, APN exerts multiple biological effects on regulating insulin resistance, inhibiting arteriosclerosis, promoting revascularisation, and modulating the immune system [10–12]. APN levels are associated with type 2 diabetes; obesity; and cardiovascular, neurodegenerative, and kidney diseases, among others [13–15]. Regarding the role of APN in promoting revascularisation and immunomodulation, we hypothesised that APN is involved in the pathogenesis of HFRS. Du et al. have studied changes in the total APN level in the plasma of patients with HFRS and found that total APN was elevated and associated with disease severity [16]. Interestingly, HMW APN has the highest binding activity, which is considered the most physiologically active form [9, 17]. HMW APN or the ratio of HMW/total APN is strongly associated with some cardiovascular and infectious diseases and could be considered sensitive biomarkers for these diseases [18–20]. Therefore, investigating the changes in the level of total and HMW APN and associated correlations with clinical parameters of HFRS is an important avenue of study. However, the level of HMW APN in the plasma of patients with HFRS and the effects of the different forms of HMW APN in HFRS pathogenesis are largely unknown.

In this study, we analysed the levels of total and HMW APN in the plasma of patients with HFRS and found that the degree of elevation in the plasma levels of total and HMW APN differed starting from the onset of fever; then, both peaked in the convalescent phase. The sustained high level of HMW APN in the convalescent phase was associated with disease severity, and the elevated level of HMW APN contributed to an increase in the total APN. These results suggested that both total and HMW APN may play a role after HTNV infection in HFRS patients.

## 2. Materials and Methods

### 2.1. Ethics Statement

The study was approved by the Institutional Review Board of the Fourth Military Medical University. All of the subjects signed an informed consent form before their blood was collected.

### 2.2. Sample Preparation

Blood samples were intravenously collected from 77 hospitalised patients with HFRS between 2013 and 2016 at the Tangdu Hospital of the Fourth Military Medical University (Xi'an, China) and from 16 healthy donors (normal controls, NC). The clinical diagnosis of HFRS was serologically confirmed by detecting specific IgM and IgG antibodies to HTNV. The plasma samples were isolated from EDTA (anticoagulant)-treated blood samples by centrifugation and cryopreserved at −80°C before analysis.

According to the diagnostic criteria from the Prevention and Treatment Strategy of HFRS described by the Ministry of Health, the People's Republic of China, patients with HFRS were classified into four clinical types: (1) mild—mild kidney damage with proteinuria ranging from “+” to “++” and no obvious oliguric period; (2) moderate—obvious symptoms of effusion (bulbar conjunctiva), uraemia, haemorrhage (skin and mucous membrane), and kidney damage with “+++” urinary protein and occurrence of significant oliguric period; (3) severe—severe effusion (bulbar conjunctiva and either pleura or peritoneum), uraemia, haemorrhage (skin and mucous membrane), and kidney damage with oliguria (urine output, 50–500 mL/day) for ≤5 days or anuria (urine output, <50 mL/day) for ≤2 days; and (4) critical—≥1 of the following symptoms during severe diseases: visceral haemorrhage, refractory shock, heart failure, pulmonary oedema, brain oedema, severe secondary infection, and severe kidney damage with oliguria (urine output, 50–500 mL/day) for >5 days, anuria (urine output, <50 mL/day) for >2 days, or a blood urea nitrogen level of >42.84 mmol/L [21, 22].

According to clinical observation, HFRS is defined by five sequential stages: febrile, hypotensive, oliguric, diuretic, and convalescent. These stages are usually classified as the acute phase (febrile, hypotensive, and oliguric stages) or the convalescent phase (diuretic and convalescent stages) [23]. In general, samples were collected at 3–6 days for the febrile or hypotension stage, 7–12 days for the oliguric stage, 13–18 days for the diuretic stage, and after 18 days for the convalescent stage. The phase within 8 days from the fever onset to the early oliguric stage was typically defined as the acute or early phase of the disease.

Patients with other kidney diseases, haematological diseases, diabetes, cardiovascular diseases, autoimmune diseases, viral hepatitis, and other liver diseases were excluded from this study.

### 2.3. Enzyme-Linked Immunosorbent Assay for the Detection of Total and HMW APN Levels in Plasma

The total APN and HMW APN levels in plasma were measured using separate sandwich enzyme-linked immunosorbent assay (ELISA) kits (R&D systems, DRP300, DHWAD0), according to the manuals. A monoclonal antibody specific for the globular domain of human APN and a monoclonal antibody specific for human HMW APN were separately precoated onto the microplates. Standards and samples were pipetted into the wells, and after washing away any unbound substances, the corresponding enzyme-linked monoclonal antibodies were added to the wells. After washing, substrate solutions were added to the wells for detection. Optical densities were determined at 450 nm with a 570 nm wavelength correction.

### 2.4. Western Blot for Analysing Plasma APN

Waki et al. have demonstrated that SDS-PAGE under nonreducing and non-heat-denaturing conditions clearly separates multimer species of adiponectin [8]. 5× sample buffer for nonreducing conditions was composed of 10% SDS, 250 mM Tris-HCl pH 6.8, and 50% glycerol. The plasma sample was mixed with 5× sample buffer and incubated for 1 hour at room temperature. The plasma proteins were separated by 4–20% SDS-PAGE and electrotransferred to a nitrocellulose membrane, after which the membrane was blocked with blocking buffer (Tris-buffered saline and 0.1% Tween 20 with 8% nonfat dry milk) for 1 h and then probed with the Human Adiponectin/Acrp30 Antibody (R&D systems, AF1065) overnight at 4°C. The membrane was developed using the Goat IgG Horseradish Peroxidase-conjugated Antibody (R&D systems, HAF019) and enhanced chemiluminescence.

### 2.5. Statistical Analysis

The analysis was performed in GraphPad Prism6 software. Continuous variables were presented as medians with corresponding interquartile ranges (IQRs). The significance of the differences between different groups was determined by the Mann–Whitney *U* test. The nonparametric Spearman correlation test was used for correlation analysis of the total and HMW APN levels with clinical parameters. *p* values (two-tailed) below 0.05 were considered statistically significant.

## 3. Results

### 3.1. Clinical Information of the Study Subjects

A total of 171 plasma samples from 77 patients with HFRS were examined, and 16 plasma samples from 16 healthy donors were assessed in this study. The information of all enrolled subjects is summarized in Table 1.

### 3.2. Dramatic Elevation of the Total and HMW APN Levels in the Plasma of Patients with HFRS

The median levels of total and HMW APN in the plasma with their IQRs are listed in Table 2. Compared with those in the normal controls, the increased percentages of the total and HMW APN levels in the plasma were 81.6% and 62.8%, respectively, in the febrile/hypotensive stage, and the elevation rate of the total APN level was 1.46-fold more than that of HMW APN. From the febrile/hypotensive stage to the diuretic/convalescent stage, the increased percentages of the total and HMW APN levels in the plasma were 44.1% and 72.1%, respectively, and the elevation rate of the HMW APN level was 1.63-fold more than that of total APN (Table 2).

**Figure 1:**
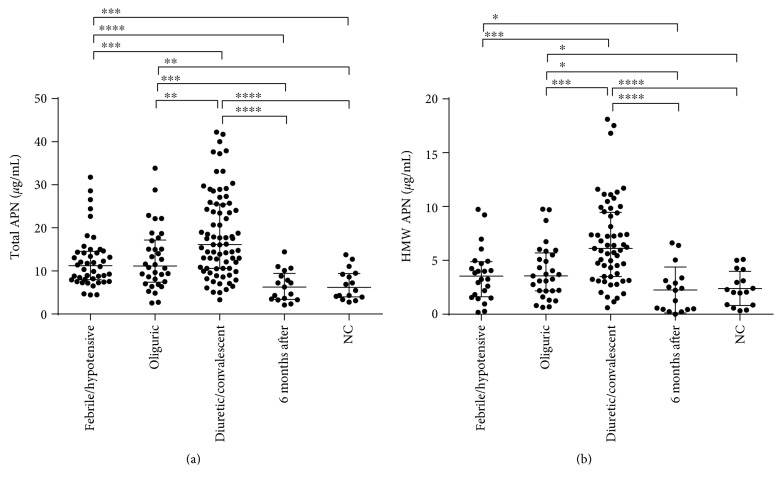
Dynamic changes of the total adiponectin (APN) and high molecular weight (HMW) APN levels in the plasma of patients with HFRS. Comparison of (a) total APN and (b) HMW APN levels in the plasma of patients with HFRS over the different stages of the disease and 6 months after discharge from the hospital. Both total APN and HMW APN levels in the plasma of patients with HFRS were generally elevated starting from the onset of fever, gradually continued to elevate during the course of the disease, and recovered to normal levels when assessed 6 months after discharge. The data were obtained from 171 plasma samples of patients with HFRS and from 16 plasma samples of healthy subjects included as normal controls (NC). The Mann–Whitney *U* test was used to determine the significance of the difference between two groups, and the black lines represent the medians with the corresponding interquartile range. ^∗^*p* < 0.05; ^∗∗^*p* < 0.01; ^∗∗∗^*p* < 0.001; ^∗∗∗∗^*p* < 0.0001.

In general, both the total APN and HMW APN levels in the patients with HFRS at different clinical stages of disease were elevated compared with those in the normal controls. The total APN level was elevated at the febrile/hypotensive stage (febrile/hypotensive versus NC, *p* < 0.001) and peaked in the diuretic/convalescent stage (diuretic/convalescent versus febrile/hypotensive, *p* < 0.001; diuretic/convalescent versus oliguric, *p* < 0.01). The median level of HMW APN was higher in the febrile/hypotensive stage of HFRS than that in the normal controls, but without statistical significance. In the oliguric stage, the HMW APN level was significantly higher than that in the normal controls (*p* < 0.05) and peaked in the diuretic/convalescent stage (diuretic/convalescent versus oliguric, *p* < 0.001). There was no significant difference in the total or HMW APN levels between the febrile/hypotensive and oliguric stages (Figure 1). Both the total APN and HMW APN levels in the plasma decreased to normal when detected 6 months after discharge from the hospital (Figure 1).

### 3.3. Dynamic Changes of Different APN Forms in the Same HFRS Individual

**Figure 2:**
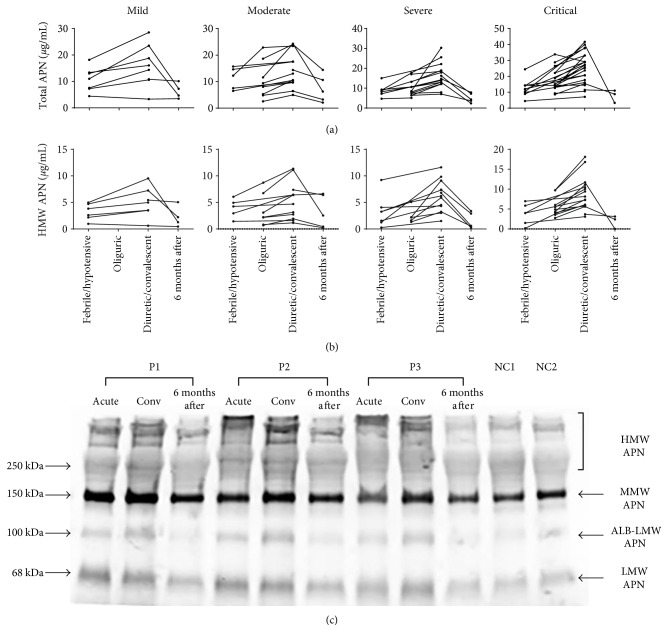
Levels of the total APN and HMW APN in plasma during the different stages of HFRS. Changes in the levels of (a) total APN and (b) HMW APN in the plasma of the same individuals from different disease severity groups are shown. (c) Under nonreducing and non-heat-denaturing conditions, a comparison of the plasma APN levels in the acute phase (the febrile, hypotensive, or oliguric stages), the convalescent phase (conv; the diuretic stage or convalescent stage), and 6 months after discharge in the same patients with HFRS and in normal controls is analysed by western blot (representative results of 3 patients with HFRS and 2 healthy subjects). Trimeric low molecular weight (LMW, 68 kDa), albumin-bound LMW (ALB-LMW, 100 kDa), hexameric middle molecular weight (MMW, 150 kDa), and HMW (>250 kDa) forms of APN are marked by black arrows. Plasma LMW plus ALB-LMW APN, MMW APN, and HMW APN levels in patients with HFRS were all elevated in the acute phase and became higher in the convalescent phase, whereas the levels of all different forms of plasma APN recovered to normal levels when measured 6 months after discharge.

The dynamic changes of the same individual in different severity groups also showed the same tendency. The plasma total and HMW APN levels in the patients with HFRS were elevated in the acute phase (febrile, hypotensive, or oliguric stage), then peaked in the convalescent phase (diuretic or convalescent stage), and recovered to normal levels 6 months after discharge (Figures 2(a) and 2(b)).

APN exists as LMW, albumin (ALB)-LMW, MMW, and HMW, which are four species of multimers in human plasma, and SDS-PAGE under nonreducing and non-heat-denaturing conditions clearly separates multimeric forms of APN [8, 9]. In the same patients with HFRS, the western blot results showed that the levels of LMW plus ALB-LMW APN, MMW APN, and HMW APN in the plasma of the patients with HFRS were all elevated in the acute phase (febrile, hypotensive, or oliguric stage) compared with those in the normal controls and then further increased in the convalescent phase. The levels of all different forms of plasma APN recovered to normal levels 6 months after discharge, which was consistent with the results of the ELISA (Figure 2(c)).

### 3.4. Plasma Total and HMW APN Levels during the Convalescent Phase Are Related to Disease Severity in Patients with HFRS

**Figure 3:**
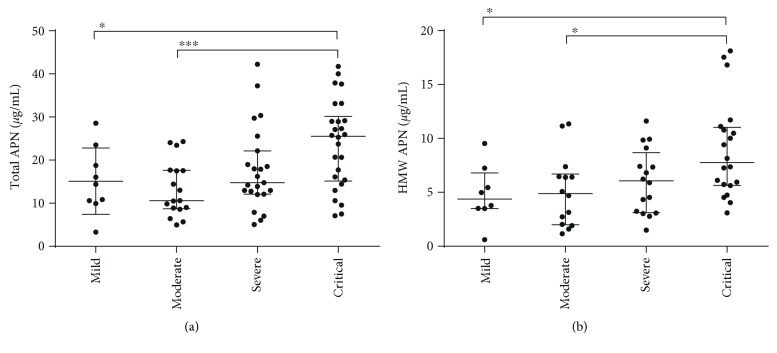
Comparison of the total APN and HMW APN levels in the plasma of patients with HFRS belonging to four disease severity groups in the convalescent phase. Both (a) total APN and (b) HMW APN levels in the plasma of critical patients were significantly higher than those of patients with mild and moderate disease at the convalescent phase. The Mann–Whitney *U* test was used to determine the significance of the differences between the two groups, and the black lines represent the medians with the corresponding interquartile range. ^∗^*p* < 0.05; ^∗∗∗^*p* < 0.001.

Next, we compared the total and HMW APN levels in the plasma of patients with HFRS delineated into different disease severity groups. During the acute phase, there was no significant difference in the total or HMW APN levels in the plasma among the four severity groups (data not shown). During the convalescent phase, the median levels of the total and HMW APN levels with their IQRs were 14.39 (10.27–21.16) *μ*g/mL and 2.60 (1.82–4.70) *μ*g/mL in patients with mild disease, 10.58 (8.75–17.64) *μ*g/mL and 3.09 (1.80–5.49) *μ*g/mL in patients with moderate disease, 14.76 (12.11–22.14) *μ*g/mL and 3.25 (1.57–4.83) *μ*g/mL in patients with severe disease, and 25.52 (15.15–30.14) *μ*g/mL and 4.17 (2.96–5.98) *μ*g/mL in patients with critical disease. Both the total and HMW APN levels in the patients with critical disease were significantly higher than those in the patients with mild or moderate disease (*p* < 0.05) (Figures 3(a) and 3(b)). However, there was no significant difference between the mild, moderate, and severe groups. Generally, the more serious the condition during the acute phase, the higher the levels of total APN and HMW APN in the plasma during the convalescent phase.

### 3.5. Plasma Total and HMW APN Levels Correlate with Clinical Parameters

**Figure 4:**
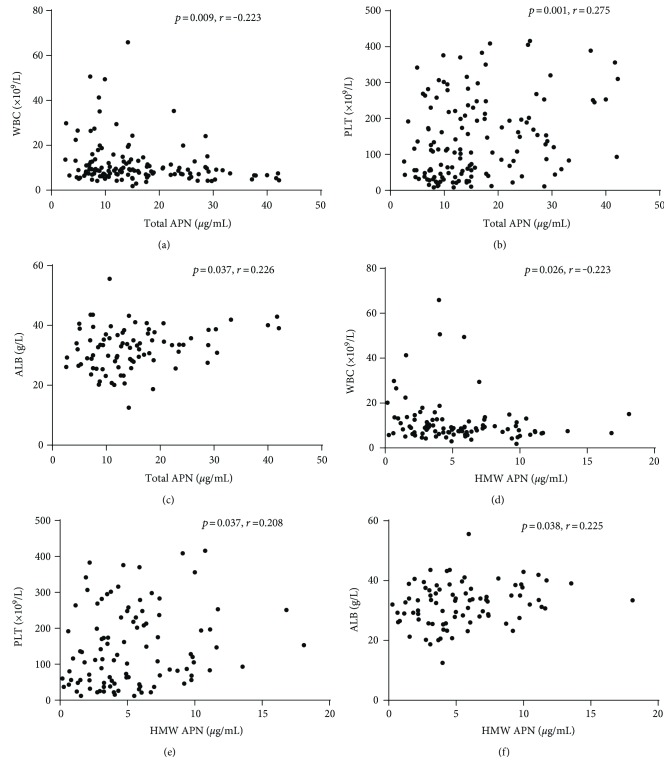
Correlation of the total APN and HMW APN levels in plasma with clinical parameters. Correlation of total APN and HMW APN in the plasma of patients with HFRS with (a, d) white blood count (WBC), (b, e) platelet count (PLT), and (c, f) serum albumin (ALB) was evaluated using the nonparametric Spearman correlation test. Each value was derived from the same patient. The *r* denotes the Spearman correlation coefficient. *p* values below 0.05 were considered statistically significant.

Total and HMW APN levels in the plasma were analysed for correlation with clinical parameters recorded on the same day as sample collection. Both total APN and HMW APN levels in the plasma negatively correlated with the white blood cell (WBC) count (Figures 4(a) and 4(d)). In contrast, the total APN and HMW APN levels in the plasma positively correlated with the platelet (PLT) count (Figures 4(b) and 4(e)) and with the serum albumin (ALB) level (Figures 4(c) and 4(f)).

## 4. Discussion

Our study first demonstrated that the total and HMW APN levels in plasma in our study cohort were elevated to different degrees starting from the onset of fever and peaked in the convalescent phase. Importantly, the sustained high levels of total and HMW APN during the convalescent phase were significantly higher in patients with critical HFRS than those in patients with mild or moderate HFRS, suggesting that APN may play an important role in the pathogenesis of HFRS.

Although we could not obtain accurate levels of LMW plus MMW APN by ELISA, we found that not only HMW APN but also LMW plus LMW-ALB and MMW APN levels were elevated during the acute phase of HFRS by western blot. All of the levels, including HMW APN, LMW plus LMW-ALB, and MMW APN, increased during the convalescent phase. Considering that there was no difference in the ratio of HMW/total APN between the patients with HFRS (median = 0.35, IQR = 0.27–0.47) and the normal controls (median = 0.34, IQR = 0.21–0.45), we concluded that LMW, MMW, and HMW APN levels were all elevated during HTNV infection and contributed to an increase in the total APN.

APN functions as a cytokine to affect inflammatory processes and immune functions [10]. Previous studies have shown that APN has an anti-inflammatory effect on ECs through the inhibition of nuclear factor-*κ*B (NF-*κ*B) signalling in vascular ECs caused by TNF-*α*-induced activation, and APN also reduces the expression of VCAM-1, E-selectin, and ICAM-1 on ECs, indicating that APN may play a role in modulating the endothelial inflammatory response [24–26]. Compared with APN-sufficient mice, APN-deficient mice had higher concentrations of plasma TNF-*α*, which supported the anti-inflammatory effect of APN [26]. The negative correlation of total and HMW APN levels in the plasma with WBC implies a potential relationship between APN concentration and leukocyte count. APN inhibits the growth of granulocyte-macrophage precursors (CFU-GM), negatively regulates granulopoiesis, and suppresses proinflammatory cytokine production by monocytes/macrophages to inhibit inflammatory processes [7, 27]. Importantly, APN promotes monocyte differentiation into anti-inflammatory M2 macrophages and acts as a direct regulator of macrophage polarisation by supporting the switch from a proinflammatory M1-like state to an anti-inflammatory M2-like state [28, 29]. In addition, APN is a negative regulator of T cell activity by inhibiting the proliferation of antigen-specific T cell lines and could also control regulatory T cell homoeostasis [30]. High levels of TNF-*α*, IL-6, and other proinflammatory cytokines are linked to more severe HFRS [4]. In our study, APN levels were found to be elevated starting from the onset of fever, indicating that the elevated levels of APN during the acute phase of HFRS may be beneficial for patients and may decrease the influence of proinflammatory cytokines on ECs.

Monomers of APN consist of an amino-terminal, collagen-like region and a carboxyl-terminal, complement factor C1q-like globular domain [30]. Considering the structural similarity of APN and C1q, some have posed a hypothetical question of whether APN can bind to the receptors of C1q-gC1qR (binding to the globular head of C1q) and cC1qR (calreticulin; interacting with both the collagen-like tail and globular head domain of C1q). APN can interact with cC1qR and its adaptor protein CD91 for the uptake and removal of apoptotic cells by macrophages, which may be crucial for preventing cell lysis and the release of proinflammatory to protect the organism from systemic inflammation [11, 31]. Therefore, elevated APN levels in patients with HFRS may be involved in the processes of inflammation control, tissue remodelling, and maintaining homeostasis via the removal of apoptotic cells.

APN can act as an endogenous antithrombotic factor to directly stimulate the production of NO in ECs, which could regulate platelet activation and cause the inhibition of platelet adhesion and aggregation [32]. Kato et al. showed that decreased APN levels contribute to enhanced thrombus formation and platelet aggregation [33]. Connolly-Andersen et al. observed increased thrombopoiesis and platelet activation contributing to intravascular coagulation and thrombocytopoenia [5]. Our study showed that both total and HMW APN levels in the plasma positively correlate with PLT counts. Therefore, the elevated APN levels may play a role in the regulation of platelet activation during HTNV infection.

Different forms of APN play different roles in biological activity. HMW APN, but not LMW APN, induces IFN-*γ* production by PBMCs from patients with chronic HCV infection [34], suggesting that HMW APN may have an antiviral role. T-cadherin protects ECs from oxidative stress-induced apoptosis [35, 36]. The MMW and HMW forms of APN, but not LMW APN, can bind to T-cadherin expressed on ECs, and HMW APN specifically suppresses EC apoptosis [37, 38]. HMW APN may be the only form of APN to interact with T-cadherin to regulate ECs and mediate revascularisation [12, 39]. Interestingly, our study demonstrated that the HMW APN level during the convalescent phase is higher in patients with more severe HFRS. Since vascular leakage in patients with mild/moderate HFRS is not obvious or is even absent, the viral load of HTNV is always undetectable and the disease is almost mitigated in the convalescent phase [40]; the high levels of HMW APN during the convalescent phase after clearing the HTNV infection may function physiologically to promote the recovery of impaired vascular ECs. This supposition is similar to our previous study that serum vascular endothelial growth factor (VEGF) is sustained at high levels during the convalescent phase in patients with more severe HFRS and associated with vascular repair [21]. In addition, total APN can upregulate the expression of VEGF via the AMP-activated protein kinase (AMPK) signalling pathway [14, 39, 41]. Thus, the sustained high levels of HMW and total APN may cooperate with VEGF to participate in repairing ECs.

The elevation rates of total and HMW APN levels in the plasma were different throughout the course of HFRS. From fever onset, the elevation rate of the total APN levels was 1.46-fold more than that of HMW APN. Oligomerisation is a significant determinant of flux across endothelial monolayers, as LMW APN is preferentially transported, and the rapid increase in absolute quantity of total APN in the plasma seems more effective at controlling fulminant inflammation [16, 17, 42]. From the febrile/hypotensive stage to the diuretic/convalescent stage, the elevation rate of the HMW APN levels was 1.63-fold more than that of total APN. Plasma LMW, MMW, and HMW APN are cleared differentially, as HMW APN is cleared the slowest and LMW is cleared the fastest [43], which may induce the difference and favour the repair of vascular ECs for the specific function of HMW APN as we discussed above. Notably, either in the acute phase or in the convalescent phase of patients with HFRS, there was no significant difference in the ratio of HMW/total APN between patients with HFRS and normal controls and the ratio change was still in the normal range.

## 5. Conclusion

In summary, the total and HMW APN levels in the plasma were elevated to different degrees after HTNV infection, and high levels were sustained during the convalescent phase. The elevation of total APN is due to the increase in LMW, MMW, and HMW APN levels, and the sustained high levels of total and HMW APN in the plasma during the convalescent phase were associated with the disease severity. These results may provide insights into understanding the roles of total and HMW APN in the pathogenesis of HFRS and the cross-talk between adipocytes and the immune system after HTNV infection.

## Conflicts of Interest

The authors declare that they have no conflicts of interest.

## Figures and Tables

**Table 1:** Characteristics of enrolled subjects in this study.

	Mild	Moderate	Severe	Critical	NC
Demographic characteristics					
Number	10	17	21	29	16
Age (years)	30 (22–46)	40 (34–55)	46 (33–58)	38 (27–56)	35 (31–45)
Male (%)	80.0	82.4	85.7	75.9	81.3
Sample number					
Febrile/hypotensive	9	7	12	16	—
Oliguric	0	9	9	16	—
Diuretic/convalescent	9	18	23	26	—
6 months after discharge	4	5	5	3	—

NC: normal controls.

Values represent medians with the corresponding interquartile range.

**Table 2:** Dynamic changes in the total adiponectin (APN) and high molecular weight (HMW) APN levels in the plasma of patients with HFRS during different disease stages.

	Total APN (*μ*g/mL)	HMW APN (*μ*g/mL)
Febrile/hypotensive	11.22 (7.98–14.56)	3.55 (1.62–4.87)
Oliguric	11.16 (7.40–17.17)	3.56 (2.17–5.69)
Diuretic/convalescent	16.17 (10.59–25.51)	6.11 (3.50–9.47)
6 months after discharge	6.25 (3.40–9.46)	2.22 (0.45–3.25)
Normal controls (NC)	6.18 (3.99–9.41)	2.18 (0.87–3.89)

Values represent medians with the corresponding interquartile range.

## References

[1] Vaheri A., Strandin T., Hepojoki J. (2013). Uncovering the mysteries of hantavirus infections. *Nature Reviews Microbiology*.

[2] Watson D. C., Sargianou M., Papa A., Chra P., Starakis I., Panos G. (2014). Epidemiology of hantavirus infections in humans: a comprehensive, global overview. *Critical Reviews in Microbiology*.

[3] Marcos-Ramiro B., Garcia-Weber D., Millan J. (2014). TNF-induced endothelial barrier disruption: beyond actin and rho. *Thrombosis and Haemostasis*.

[4] Hepojoki J., Vaheri A., Strandin T. (2014). The fundamental role of endothelial cells in hantavirus pathogenesis. *Frontiers in Microbiology*.

[5] Connolly-Andersen A. M., Sundberg E., Ahlm C. (2015). Increased thrombopoiesis and platelet activation in hantavirus-infected patients. *The Journal of Infectious Diseases*.

[6] Magkos F., Sidossis L. S. (2007). Recent advances in the measurement of adiponectin isoform distribution. *Current Opinion in Clinical Nutrition and Metabolic Care*.

[7] Crawford L. J., Peake R., Price S., Morris T. C., Irvine A. E. (2010). Adiponectin is produced by lymphocytes and is a negative regulator of granulopoiesis. *Journal of Leukocyte Biology*.

[8] Waki H., Yamauchi T., Kamon J. (2003). Impaired multimerization of human adiponectin mutants associated with diabetes. Molecular structure and multimer formation of adiponectin. *The Journal of Biological Chemistry*.

[9] Hada Y., Yamauchi T., Waki H. (2007). Selective purification and characterization of adiponectin multimer species from human plasma. *Biochemical and Biophysical Research Communications*.

[10] Tilg H., Moschen A. R. (2006). Adipocytokines: mediators linking adipose tissue, inflammation and immunity. *Nature Reviews Immunology*.

[11] Ouchi N., Parker J. L., Lugus J. J., Walsh K. (2011). Adipokines in inflammation and metabolic disease. *Nature Reviews Immunology*.

[12] Parker-Duffen J. L., Nakamura K., Silver M. (2013). T-cadherin is essential for adiponectin-mediated revascularization. *The Journal of Biological Chemistry*.

[13] Fasshauer M., Bluher M. (2015). Adipokines in health and disease. *Trends in Pharmacological Sciences*.

[14] Yang Y., Hu W., Jiang S. (2015). The emerging role of adiponectin in cerebrovascular and neurodegenerative diseases. *Biochimica et Biophysica Acta*.

[15] Jia T., Carrero J. J., Lindholm B., Stenvinkel P. (2012). The complex role of adiponectin in chronic kidney disease. *Biochimie*.

[16] Du H., Bai X., Lian J. (2016). Changes in plasma adiponectin concentrations in patients with hemorrhagic fever with renal syndrome: an observational prospective study. *Medicine (Baltimore)*.

[17] Esmaili S., Xu A., George J. (2014). The multifaceted and controversial immunometabolic actions of adiponectin. *Trends in Endocrinology and Metabolism*.

[18] Wang Y., Zheng A., Yan Y. (2014). Association between HMW adiponectin, HMW-total adiponectin ratio and early-onset coronary artery disease in Chinese population. *Atherosclerosis*.

[19] Omar F., Dave J. A., King J. A., Levitt N. S., Pillay T. S. (2014). High molecular weight (HMW): total adiponectin ratio is low in hiv-infected women receiving protease inhibitors. *BMC Clinical Pathology*.

[20] Sulistyoningrum D. C., Gasevic D., Lear S. A., Ho J., Mente A., Devlin A. M. (2013). Total and high molecular weight adiponectin and ethnic-specific differences in adiposity and insulin resistance: a cross-sectional study. *Cardiovascular Diabetology*.

[21] Ma Y., Liu B., Yuan B. (2012). Sustained high level of serum VEGF at convalescent stage contributes to the renal recovery after HTNV infection in patients with hemorrhagic fever with renal syndrome. *Clinical & Developmental Immunology*.

[22] Liu Z., Zhao Q., Han Q., Gao M., Zhang N. (2008). Serum thrombospondin-1 is altered in patients with hemorrhagic fever with renal syndrome. *Journal of Medical Virology*.

[23] Zhang Y., Zhang C., Zhuang R. (2015). IL-33/ST2 correlates with severity of haemorrhagic fever with renal syndrome and regulates the inflammatory response in Hantaan virus-infected endothelial cells. *PLoS Neglected Tropical Diseases*.

[24] Kobashi C., Urakaze M., Kishida M. (2005). Adiponectin inhibits endothelial synthesis of interleukin-8. *Circulation Research*.

[25] Ouchi N., Kihara S., Arita Y. (1999). Novel modulator for endothelial adhesion molecules: adipocyte-derived plasma protein adiponectin. *Circulation*.

[26] Maeda N., Shimomura I., Kishida K. (2002). Diet-induced insulin resistance in mice lacking adiponectin/ACRP30. *Nature Medicine*.

[27] Yokota T., Oritani K., Takahashi I. (2000). Adiponectin, a new member of the family of soluble defense collagens, negatively regulates the growth of myelomonocytic progenitors and the functions of macrophages. *Blood*.

[28] Lovren F., Pan Y., Quan A. (2010). Adiponectin primes human monocytes into alternative anti-inflammatory M2 macrophages. *American Journal of Physiology Heart and Circulatory Physiology*.

[29] Ohashi K., Parker J. L., Ouchi N. (2010). Adiponectin promotes macrophage polarization toward an anti-inflammatory phenotype. *The Journal of Biological Chemistry*.

[30] Procaccini C., De Rosa V., Galgani M. (2013). Role of adipokines signaling in the modulation of T cells function. *Frontiers in Immunology*.

[31] Takemura Y., Ouchi N., Shibata R. (2007). Adiponectin modulates inflammatory reactions via calreticulin receptor-dependent clearance of early apoptotic bodies. *The Journal of Clinical Investigation*.

[32] Ekmekci H., Ekmekci O. B., Erdine S. (2009). Effects of serum homocysteine and adiponectin levels on platelet aggregation in untreated patients with essential hypertension. *Journal of Thrombosis and Thrombolysis*.

[33] Kato H., Kashiwagi H., Shiraga M. (2006). Adiponectin acts as an endogenous antithrombotic factor. *Arteriosclerosis, Thrombosis, and Vascular Biology*.

[34] Palmer C., Hampartzoumian T., Lloyd A., Zekry A. (2008). A novel role for adiponectin in regulating the immune responses in chronic hepatitis C virus infection. *Hepatology*.

[35] Joshi M. B., Philippova M., Ivanov D., Allenspach R., Erne P., Resink T. J. (2005). T-cadherin protects endothelial cells from oxidative stress-induced apoptosis. *The FASEB Journal*.

[36] Andreeva A. V., Han J., Kutuzov M. A., Profirovic J., Tkachuk V. A., Voyno-Yasenetskaya T. A. (2010). T-cadherin modulates endothelial barrier function. *Journal of Cellular Physiology*.

[37] Hug C., Wang J., Ahmad N. S., Bogan J. S., Tsao T. S., Lodish H. F. (2004). T-cadherin is a receptor for hexameric and high-molecular-weight forms of Acrp30/adiponectin. *Proceedings of the National Academy of Sciences of the United States of America*.

[38] Kobayashi H., Ouchi N., Kihara S. (2004). Selective suppression of endothelial cell apoptosis by the high molecular weight form of adiponectin. *Circulation Research*.

[39] Shen L., Miao J., Yuan F. (2013). Overexpression of adiponectin promotes focal angiogenesis in the mouse brain following middle cerebral artery occlusion. *Gene Therapy*.

[40] Yi J., Xu Z., Zhuang R. (2013). Hantaan virus RNA load in patients having hemorrhagic fever with renal syndrome: correlation with disease severity. *The Journal of Infectious Diseases*.

[41] Ohashi K., Ouchi N., Sato K. (2009). Adiponectin promotes revascularization of ischemic muscle through a cyclooxygenase 2-dependent mechanism. *Molecular and Cellular Biology*.

[42] Rutkowski J. M., Halberg N., Wang Q. A., Holland W. L., Xia J. Y., Scherer P. E. (2014). Differential transendothelial transport of adiponectin complexes. *Cardiovascular Diabetology*.

[43] Halberg N., Schraw T. D., Wang Z. V. (2009). Systemic fate of the adipocyte-derived factor adiponectin. *Diabetes*.

